# Medical clowning in hospitalized children: a meta-analysis

**DOI:** 10.1007/s12519-023-00720-y

**Published:** 2023-04-14

**Authors:** Rim Kasem Ali Sliman, Noam Meiri, Giora Pillar

**Affiliations:** 1 Department of Pediatrics, Carmel Medical Center, Carmel Hospital, 7 Michal St., 3436212 Haifa, Israel; 2 Technion Faculty of Medicine, Haifa, Israel

**Keywords:** Anxiety, Medical clown, Pain, Pediatric patients

## Abstract

**Background:**

Medical clowning has been proven effective in reducing pain, anxiety, and stress in many sporadic, usually small-scale studies. Our meta-analysis aims to evaluate the efficiency of medical clowns in reducing pain and anxiety in hospitalized pediatric patients and their parents in different medical fields.

**Methods:**

A thorough literature search was conducted from different databases, and only randomized controlled trials (RCTs) were included with children aged 0 to 18 years old. A total of 18 studies were included, and statistical analysis was performed on the combined data.

**Results:**

A total of 912 children (14 studies) showed significantly reduced anxiety when procedures were performed with a medical clown compared with the controls (− 0.76 on anxiety score, *P* < 0.001). Preoperative anxiety was lower in 512 children (nine studies) with clown interventions than in the controls (− 0.78, *P* < 0.001). The pain scale was completed by 338 participants (six studies), indicating a trend toward reduced pain during procedures performed while the clown was acting compared to controls (− 0.49, *P* = 0.06). In addition, medical clown significantly (− 0.52, *P* = 0.001) reduced parental anxiety in 489 participants in ten studies; in six of the ten studies, with a total of 380 participants, medical clown significantly reduced parental preoperative anxiety (*P* = 0.02).

**Conclusion:**

Medical clowns have substantial positive and beneficial effects on reducing stress and anxiety in children and their families in various circumstances in pediatrics.

**Supplementary Information:**

The online version contains supplementary material available at 10.1007/s12519-023-00720-y.

## Background

In the last two decades, medical clowning has changed its role enormously from a “sideshow” in the hospital corridors to an active and meaningful role in medical procedures and therapy [1, 2]. In recent years, the use of medical clowns (MC) as part of the legitimate medical staff has received increasing attention from health professionals [3, 4]. Hospitalization in general, and in children in particular, may have negative consequences both acutely and chronically on emotional, behavioral, cognitive, and educational development [2, 4–6]. Distress and anxiety are widespread in children undergoing medical procedures [7]. MC interventions may distract children from pain-inducing sources, which may reduce psychological distress and pain in these children and their parents, in addition to increasing their cooperation during medical procedures [8, 9].

MCs are often professional actors trained explicitly for this job and equipped with various skills and tools (music, poetry, magic, etc.). In addition, the increased presence of MCs in pediatric departments, along with their assistance in medical procedures, has shown promising outcomes [1, 4, 10].

Most studies published thus far in this area incorporated a relatively small number of patients, we planned this meta-analysis to power up and combine studies to compare the clinical utility of hospital clowns to the standard of care without MC intervention to alleviate anxiety and pain in various procedures [5]. Furthermore, we assessed the extent of parental anxiety in studies that targeted this. We also studied the effect of clown therapy, specifically preoperatively, on children’s and parents’ anxiety.

## Methods

### Data collection

A thorough literature search was conducted and was completed in November 2019. The primary databases used were PubMed (Medline), Cochrane, Embase, and Google Scholar.

The keywords used for the search were MC, clown therapy, clowns, and pediatrics/children. After the initial database was created, the authors performed a further fundamental search for additional relevant studies that were not included in the primary set. Following these two steps, the database was searched for the following outcome keywords: anxiety, parental anxiety, and pain.

### Eligibility criteria

1. Randomized controlled trials.
2. Children aged 0 to 18 years old who were either admitted to the hospital or referred to a hospital-affiliated emergency room (ER).
3. Participants underwent at least one potentially painful/stressful procedure, such as blood drawing, intravenous cannulation, minor surgeries under anesthesia, or skin prick test, in the ER/pediatric ward.
4. Primary intervention by MC and a control group. Outcomes of anxiety/pain were assessed quantitatively and not only qualitatively.

### Study procedures

Two authors performed independent selection for the inclusion of studies, and only those chosen by both were included. A data extraction form was created, and two authors independently extracted the following data from each eligible study: study location, year, methods, participants, study interventions, and outcomes. Disagreement between the authors was resolved through discussion.

The extracted data were analyzed using non-Cochrane mode in RevMan5.3 software. The risk of bias was assessed by Cochrane collaboration’s tool for assessing the risk of bias. The judgment was categorized into low, high, or unclear risk of bias. SMD (standardized mean differences) was chosen as a measure of pooled results considering the variability observed in the measuring scales for continuous outcomes. A 95% confidence interval (CI) was used to represent the deviation from the point estimate for both the individual studies and the pooled estimate.

## Results

**Fig. 1:**
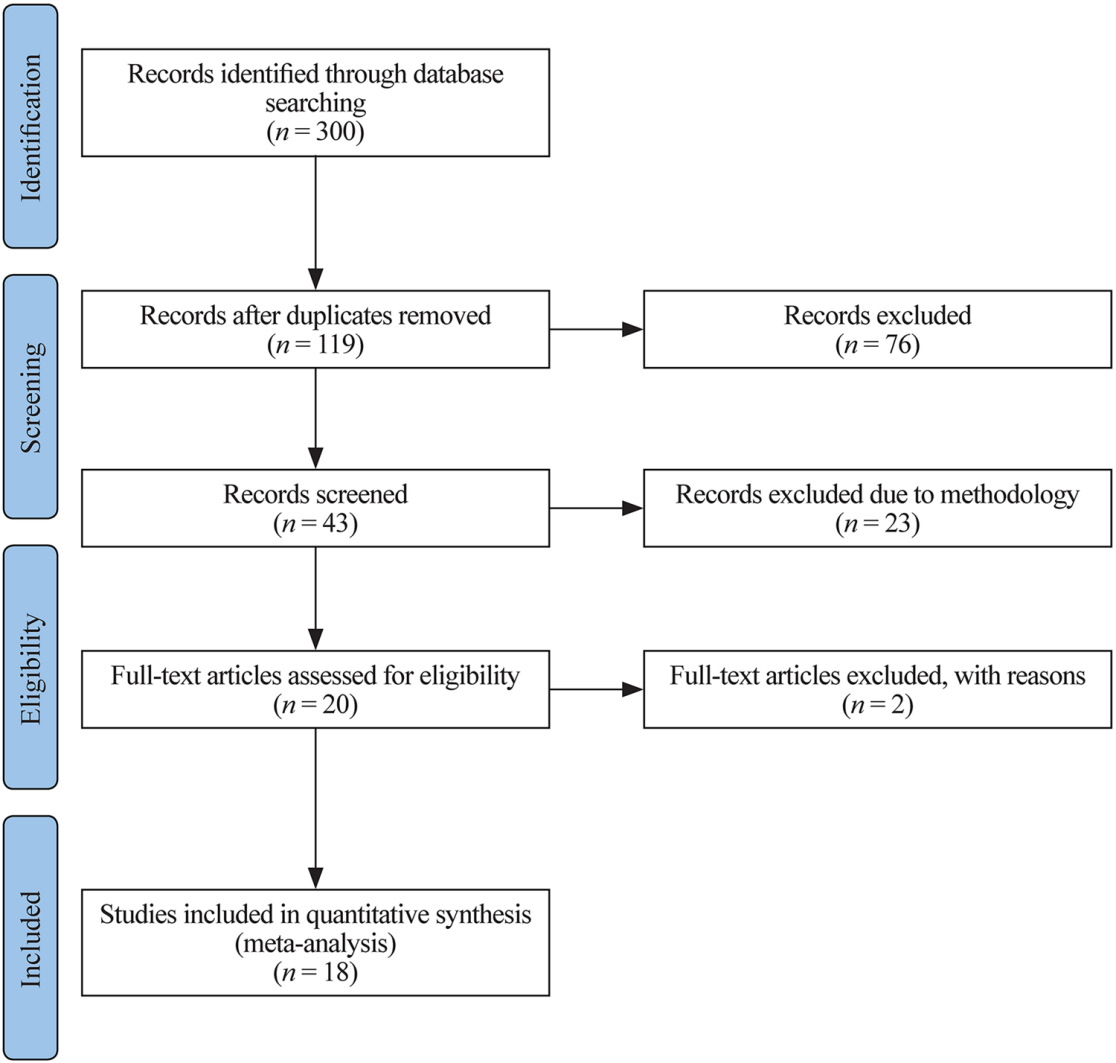
Study flow chart. A total of 119 studies were obtained with the search strategy, of which 18 were included in the meta-analysis

**Fig. 2:**
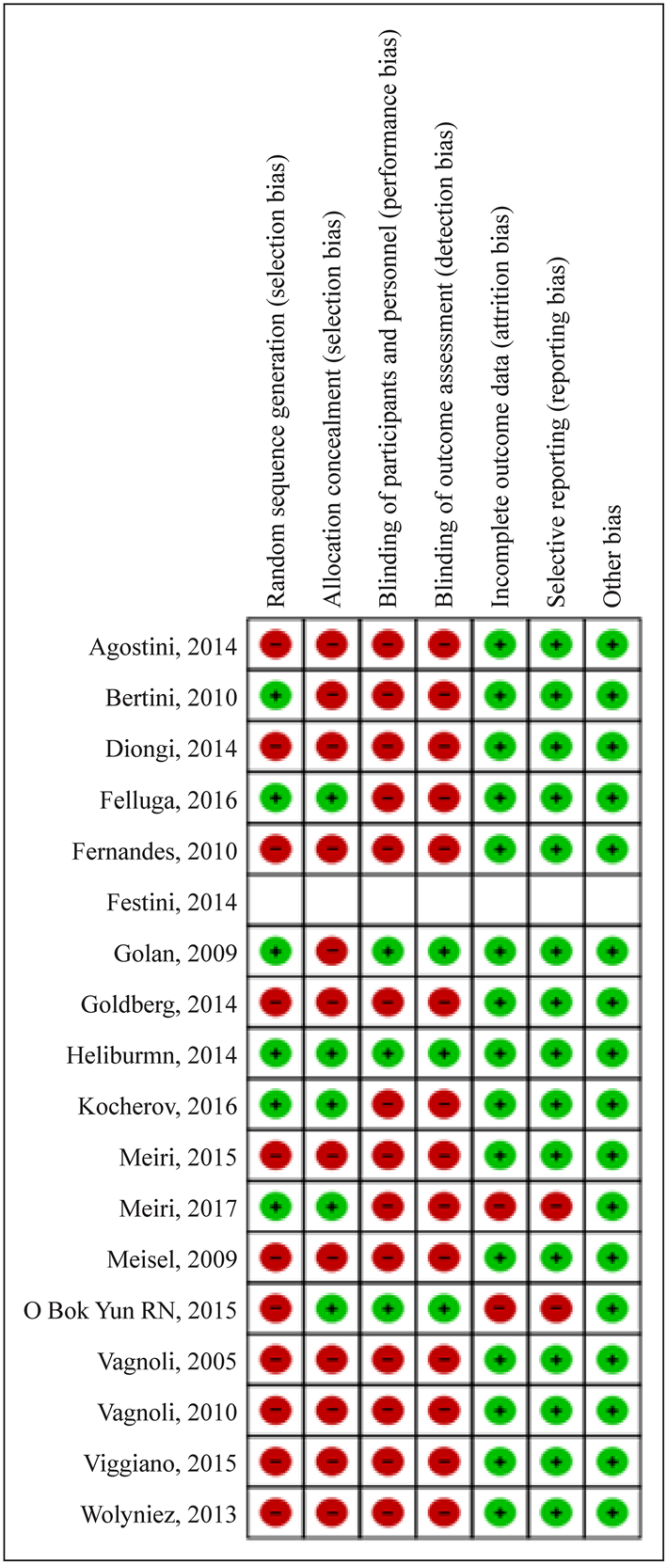
Summary of risk of bias of the included eighteen studies. Red circle with minus symbol indicates the absence of reporting specific elements by the authors while the green circle with a plus symbol indicates reporting of the same. The report by Festini et al. [13] was a conference abstract, and none of the elements of risk of bias could be assessed [5]. As seen in most reviewed papers, there are some potential biases that should be kept in mind when assessing the reported data

A total of 119 studies were initially obtained from the electronic databases; 76 studies did not meet the inclusion criteria in the first pass and were excluded. Of the remaining 43 studies carefully evaluated, only 18 studies met all inclusion criteria and were eligible to be included in the present meta-analysis (Fig. 1). The risk of bias of the studies included in the meta-analysis was assessed as described in Fig. 2. Publication bias was evaluated for the following outcomes: the extent of anxiety experienced by children (Supplementary Fig. 1) and the pain felt by children (Supplementary Fig. 2). No bias were observed by the symmetrical distribution of the effect estimates of individual studies. Publication bias was also assessed for parental anxiety, for which there was one relatively exceptional result from the rest of the results (SMD − 1.81). However, it was still within the SMD range of − 2 to + 2 and included in the statistical analysis (Supplementary Fig. 3).

### Primary outcomes

#### Effect of medical clowning on children’s anxiety

**Fig. 3:**
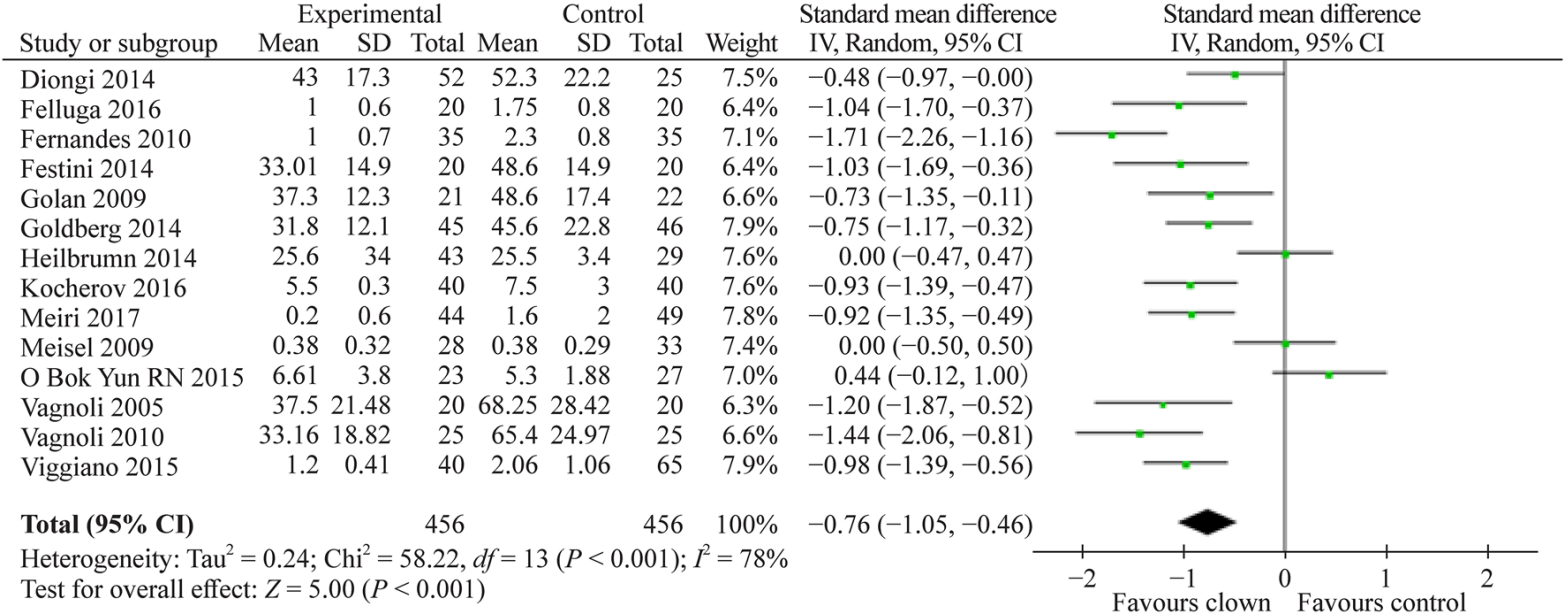
Forest plot of anxiety score with clown therapy intervention compared to standard of care without clown intervention. The pooled estimate favoring clown therapy observed a statistically significant reduction in the anxiety score. *SD* standard deviation, *CI* confidence interval

A total of 14 studies [4, 9–21] that included 912 children compared the effect of MC therapy on children’s anxiety compared to the control group. Nine studies used a modified Yale Preoperative Anxiety Scale (m-YPAS). The others used either the facial affective scale, Likert scale, child surgery worries questionnaire, or children’s anxiety and pain scales (CAPS ANXIETY) to assess children’s anxiety. The pooled SMD (95% CI) for child anxiety score was − 0.76 (− 1.05, − 0.45), favoring clown therapy (Fig. 3). This result was highly statistically significant (*P* < 0.001).

#### The effect of medical clowning on children’s pain

**Fig. 4:**
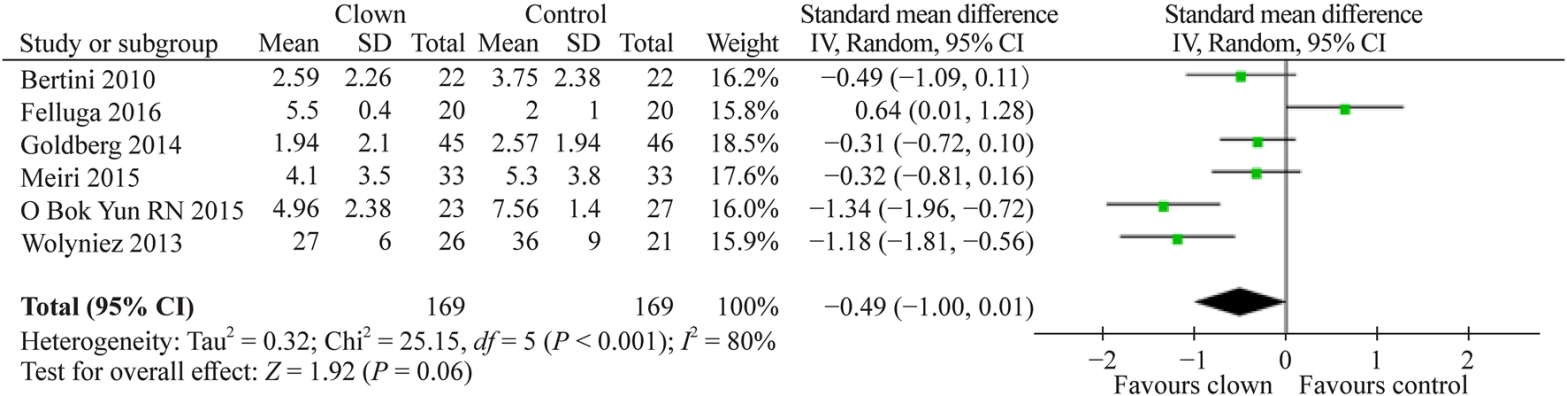
Forest plot of pain score with clown therapy intervention compared to standard of care without clown intervention. On average, of the 338 participants, the SMD of the pain score was −0.49 with clown intervention compared with standard care but without statistical significance (*P* = 0.06). *SD* standard deviation, *CI* confidence interval

The effect of MC on children’s pain during invasive procedures has been reported in six papers. Six studies [6, 9, 11, 14, 22, 23] compared the pain felt by children during procedures such as intravenous (IV) cannulation, blood drawing, minor surgery, prick skin test, hospitalization or other painful procedures in the ER between clown intervention and control. Since the number of papers assessing pain under MC is too small to discuss various pain/procedures individually, for the purpose of the current meta-analysis, we thought it is more appropriate to group them. A total of 338 participants were included. Four studies used a visual analog scale (VAS), and two employed the numerical rating scale (NRS) and/or the Wong-Baker pain scale. Figure 4 depicts the forest plot of changes in pain score. The pooled SMD (95% CI) for child pain score was − 0.49, favoring clown (−1.00, 0.01), albeit with only (*P* = 0.06).

#### Effect of medical clowning on the extent of anxiety experienced by children preoperatively

**Fig. 5:**
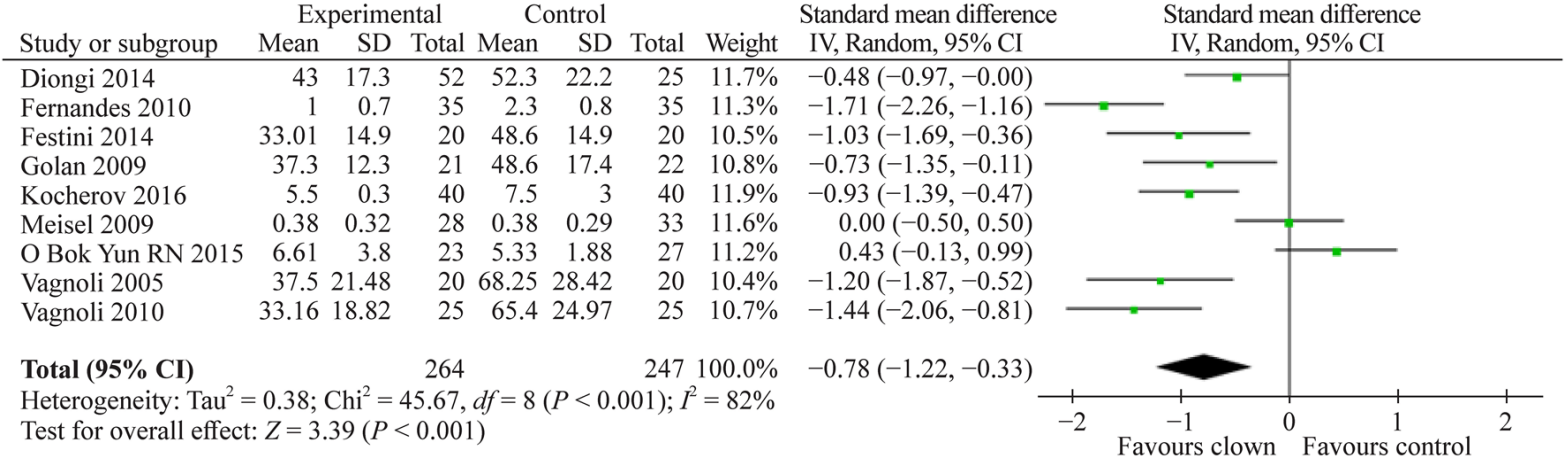
Forest plot of anxiety score preoperatively with clown therapy intervention compared to standard of care without clown intervention. A statistically significant reduction in the anxiety score preoperatively was observed, with the pooled estimate favoring the utilization of clown therapy before surgery. *SD* standard deviation, *CI* confidence interval

Nine [9, 10, 12, 13, 16, 18–21] of the 14 studies assessed anxiety in children preoperatively. A total of 511 study participants were pulled together from these studies. The pooled SMD (95% CI) for children's anxiety experienced before surgery was − 0.78 (− 1.22, − 0.33), favoring clown interventions compared to controls (*P* < 0.001) (Fig. 5).

### Secondary outcomes

#### Effect of medical clowning on parental anxiety

**Fig. 6:**
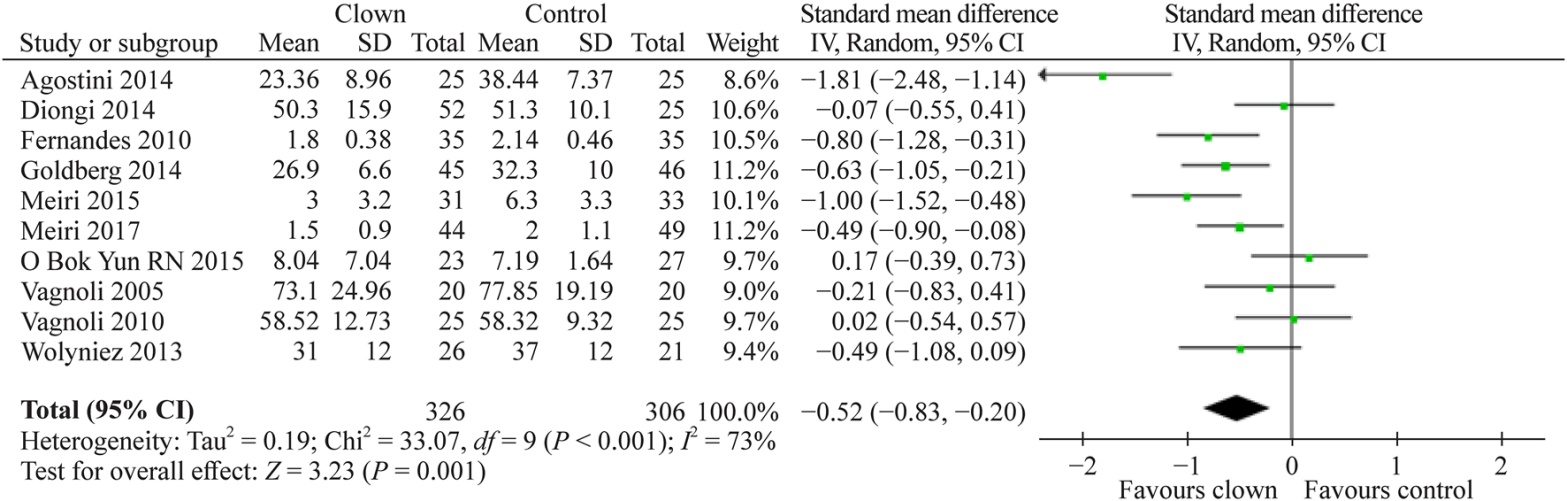
Forest plot of state anxiety experienced by parents with clown therapy intervention compared to controls without clown therapy intervention. A significant anxiety reduction was observed among parents with clown therapy intervention compared to the standard of care without clown therapy intervention. *SD* standard deviation, *CI* confidence interval

A total of 10 studies [4, 6, 9, 10, 12, 14, 18, 21, 22, 24] compared the effect of MC intervention with the standard of care without clown intervention on parental anxiety. A total of 489 study participants were assessed. The pooled SMD (95% CI) for parental state anxiety score was − 0.52 (− 0.83, − 0.20) (*P* = 0.001) (Fig. 6).

#### Effect of medical clowning on preoperative parental anxiety

**Fig. 7:**
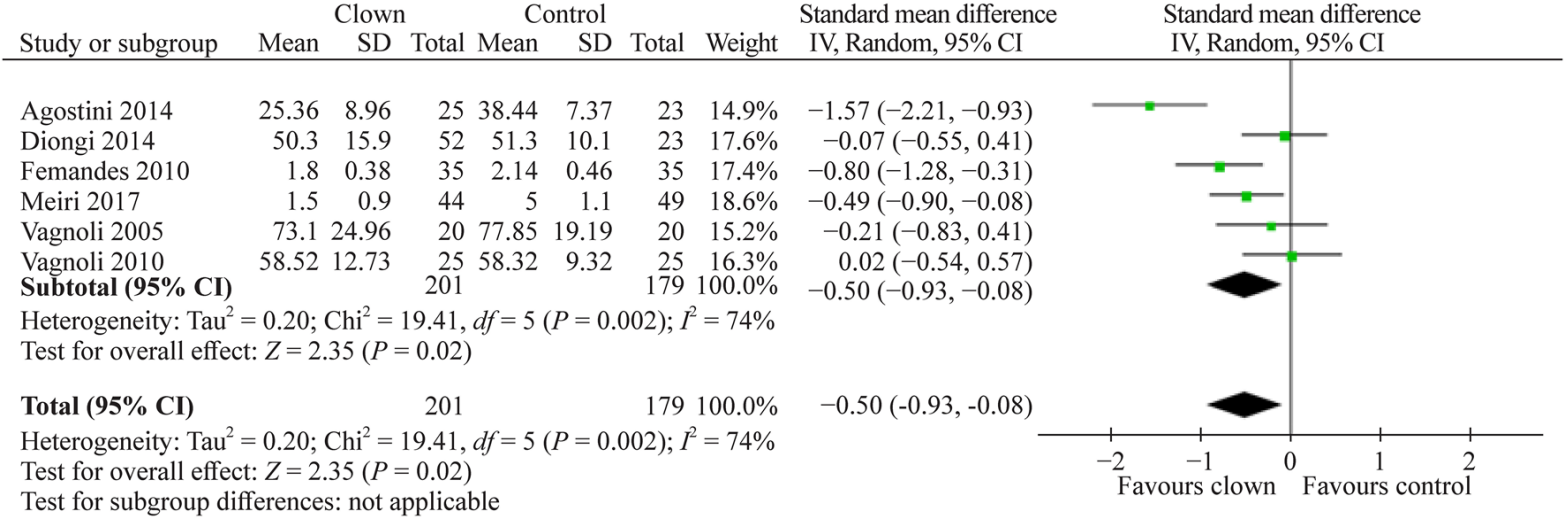
Forest plot of state anxiety experienced by parents preoperatively with clown therapy intervention compared to control without clown therapy intervention. A significant anxiety reduction was observed among parents preoperatively with clown therapy intervention compared to the standard of care without clown therapy intervention. *SD* standard deviation, *CI* confidence interval

Six studies [4, 10, 12, 18, 21, 24] of the ten studies that compared the effect of clown therapy intervention with the standard of care without clown intervention on parental anxiety assessed the extent of parental anxiety preoperatively with clown intervention in comparison to control. A total of 380 study participants were evaluated. The pooled SMD (95% CI) for parental state anxiety score was − 0.50 (− 0.93, − 0.08), favoring clown intervention over standard care (*P* = 0.02, Fig. 7).

## Discussion

The current meta-analysis indicates a substantial novel change in pediatric attitudes toward fear, stress, and pain by incorporating MC into the field of pediatrics. Our meta-analysis showed a strong and significant advantage for MC in reducing children's and parents' stress in various clinical circumstances, despite only an association that has been found between MC and reducing pain. Nevertheless, the causal link needs to be confirmed. Clearly, the field of medical clowning is evolving and growing, and more studies and data are warranted to better understand the high potential embedded in this area.

Summarizing our meta-analysis results, as can be evident from Figs. 3, 4, 5, 6, 7, the vast majority of the studies and the overall meta-analysis results indicate that MC significantly reduces the anxiety experienced by children and parents in both potentially painful procedures such as blood drawing or line insertion and potentially more painful/stressful procedures such as minor surgeries. Another finding is that MC may reduce pain during various medical procedures, although this effect was not statistically significant in our meta-analysis. This concept is of great interest and sheds light on the mechanism of improvement by the clown. Since the pain was not substantially reduced, but anxiety and crying were reduced, it indicates that in children, the fear or anxiety toward a procedure is often more pronounced than the actual pain of the procedure. In one study by Mortamet et al., the authors described that humor relieved fear and anxiety surrounding painful and invasive procedures in pediatric patients. Another study by Finlay et al. described that clowns help children adapt to their hospital surroundings and can distract and demystify painful or frightening procedures through "doses of fun" to complement traditional clinical interventions. In addition, humor may also help improve the healthcare professional-patient relationship and can act as an "ice breaker" in certain circumstances [25–27]. Another study strengthens the same concept and presents that humor suppresses patient and parent anxiety and creates a lasting beneficial effect for hospital staff. Humor also cultivates teamwork, improves morale and motivation, increases productivity, relaxes people, enhances problem-solving abilities, and creates a positive work culture with greater job satisfaction [27, 28]. The magnitude of the anxiety reduction was large with children and moderate with parents, but in both cases, it was highly statistically significant. Other systematic reviews also strengthen the results of our meta-analysis that the use of MCs in children undergoing potentially anxiety-provoking procedures seems to decrease not only children’s but also parents’ anxiety and stress levels [5, 7].

Another important issue is the cost of involving the MC in the pediatric department. In different studies, they stated that in total, the costs were reduced whenever medical clowns were used. Kocherov et al. stated that the use of MCs facilitates a reduction in patient preoperative anxiety and shortens the overall hospital stay, therefore significantly reducing overall medical cost and allowing for a greater number of patients to be cared for per unit of time [16]. Another study demonstrated that the cost of the clown is less than the total cost of using an anesthesiologist and the drugs required to sedate the patient for this kind of procedure, and some doctors noted that further investment in more medical clowns could potentially save hospital money in this respect [29]. Nevertheless, our study emphasizes the positive effect of MCs on many medical procedures and therapies. Increasing the knowledge of clown therapy may improve medical care.

This study has several limitations. First, in our study, we only included RCTs and excluded qRCTs, which may be associated with selection bias based on selective allocation [7]. Second, although we selected only RCTs, the controls were always "usual treatment" and not placebo. It is tremendously difficult to generate a placebo control for clown intervention, and in fact, none of the reviewed studies did that.

In conclusion, MC substantially has beneficial effects on children and their families in various circumstances in pediatrics. We recommend that hospitals put effort into incorporating MC into their pediatric departments. The data showed their ability to reduce stress and anxiety and, in some cases, improve outcomes. However, high-quality RCTs are needed to collect further data to better define which children and families and under which conditions and procedures the patients will mostly benefit from MC.

### Supplementary Information

Below is the link to the electronic supplementary material.

Supplementary Fig. 1 Assessment of publication bias for the anxiety score in children by Egger’s funnel plot. No bias was detected, as evidenced by the symmetrical distribution of the effect estimates of individual studies. *SE* standard error, *SMD* standard mean difference

Supplementary Fig. 2 Assessment of publication bias for the pain felt score in children by Egger’s funnel plot. No bias was detected, as evidenced by the symmetrical distribution of the effect estimates of individual studies. *SE* standard error, *SMD* standard mean difference

Supplementary Fig. 3 Assessment of publication bias for the extent of parental anxiety score by Egger’s funnel plot. One study was a relative outlier yet within the range of − 2 to + 2 SMD and thus included in the statistical analysis. *SE* standard error, *SMD* standard mean difference

## Data Availability

Raw data for this study (i.e. specific data extracted from each publication are available upon request).
