# Supplementary figures and images for: Medical clowning in hospitalized children: a meta-analysis
Source: World J Pediatr. 2023 Apr 14;19(11):1055–61. doi: 10.1007/s12519-023-00720-y (PMC10533584; doi:10.1007/s12519-023-00720-y)

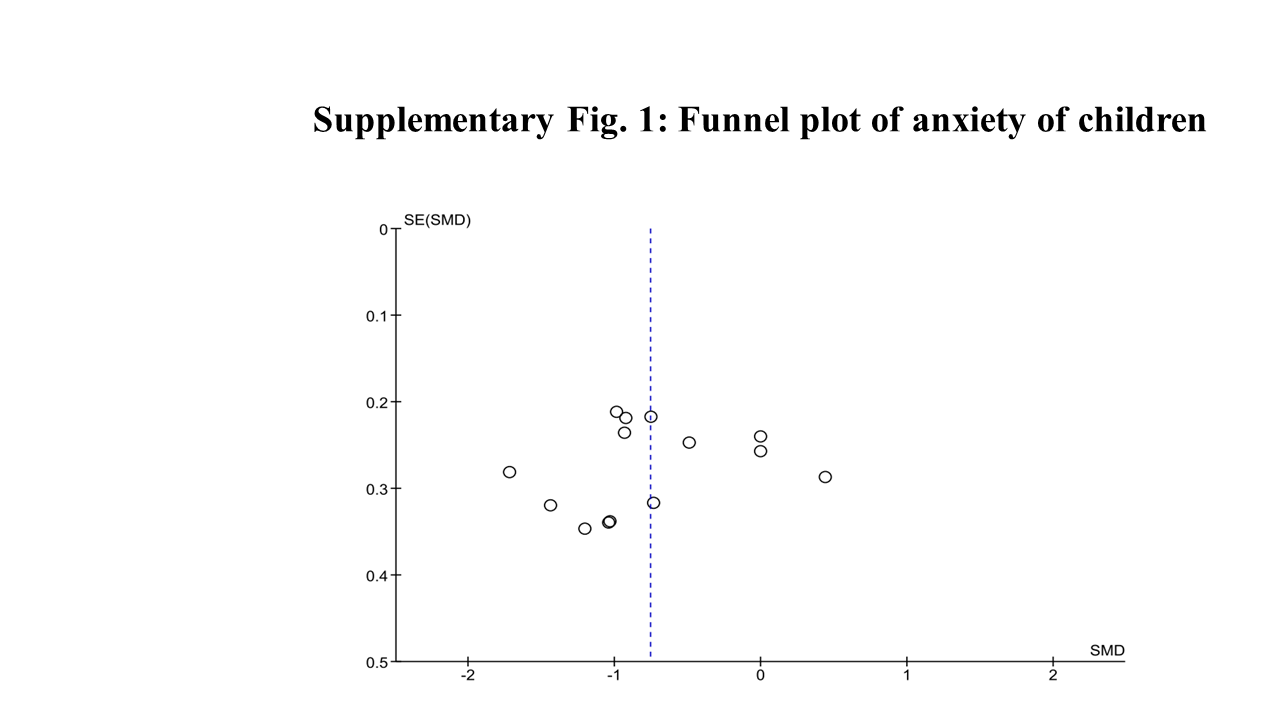

Supplement: Supplementary file 1 — Supplementary Fig. 1 Assessment of publication bias for the anxiety score in children by Egger’s funnel plot. No bias was detected, as evidenced by the symmetrical distribution of the effect estimates of individual studies. SE standard error, SMD standard mean difference [file 12519_2023_720_MOESM1_ESM.tif]

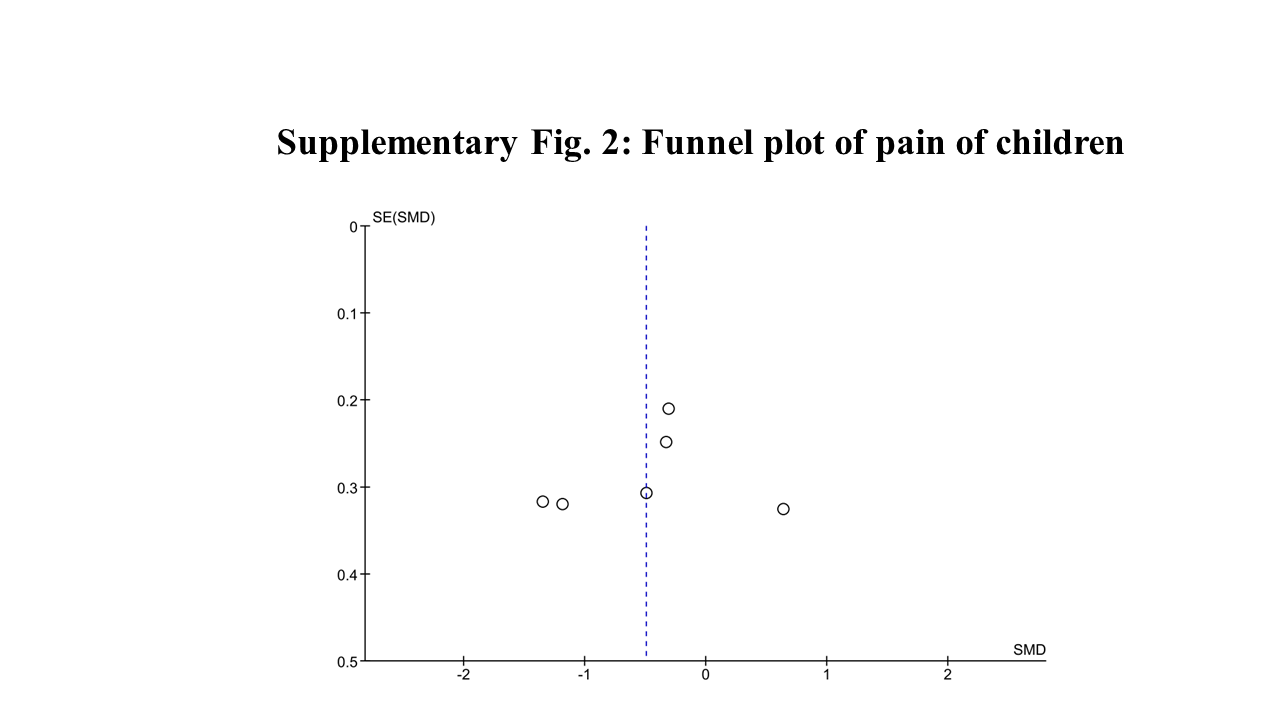

Supplement: Supplementary file 2 — Supplementary Fig. 2 Assessment of publication bias for the pain felt score in children by Egger’s funnel plot. No bias was detected, as evidenced by the symmetrical distribution of the effect estimates of individual studies. SE standard error, SMD standard mean difference [file 12519_2023_720_MOESM2_ESM.tif]

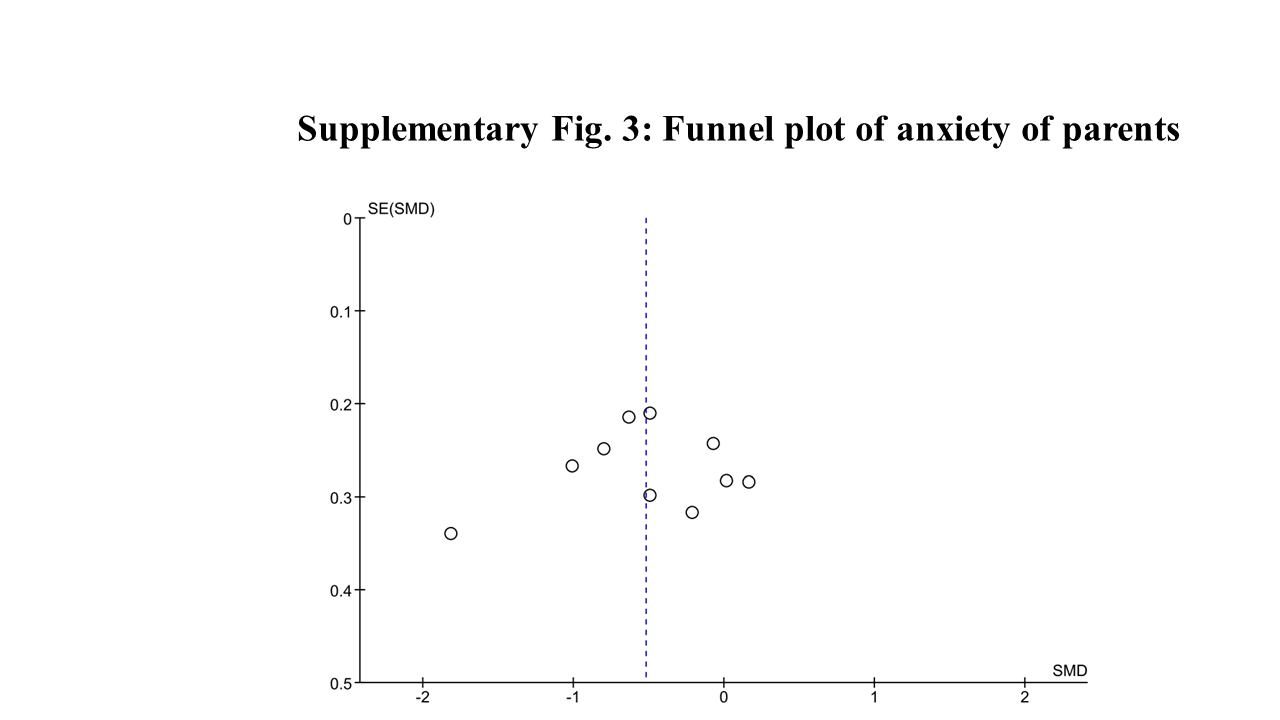

Supplement: Supplementary file 3 — Supplementary Fig. 3 Assessment of publication bias for the extent of parental anxiety score by Egger’s funnel plot. One study was a relative outlier yet within the range of − 2 to + 2 SMD and thus included in the statistical analysis. SE standard error, SMD standard mean difference [file 12519_2023_720_MOESM3_ESM.tif]
